# Esophageal epiphrenic diverticulum treated with laparoscopic surgery in a patient with systemic sclerosis: A rare case report

**DOI:** 10.1016/j.ijscr.2023.108136

**Published:** 2023-04-07

**Authors:** Ryuichi Asai, Yoshihiro Tanaka, Yuta Sato, Seito Fujibayashi, Masahide Endo, Nobuhisa Matsuhashi

**Affiliations:** Department of Gastroenterological Surgery and Pediatric Surgery, Graduate School of Medicine, Gifu University School of Medicine, 1-1 Yanagido, Gifu City 501-1194, Japan

**Keywords:** Esophageal epiphrenic diverticulum, Systemic sclerosis, Laparoscopic surgery, Diverticulectomy, Heller myotomy

## Abstract

**Introduction and importance:**

Systemic sclerosis is a disease characterized by autoimmune inflammation, fibrosis of the skin and internal organs, and vasculopathy. Diverticula found in the intestines are a common feature in patients with systemic sclerosis, but esophageal epiphrenic diverticulum is extremely rare. We present a rare case of esophageal epiphrenic diverticulum treated with laparoscopic diverticulectomy and Heller myotomy in a patient with systemic sclerosis.

**Case presentation:**

A 73-year-old woman had been treated with prednisolone for diffuse systemic sclerosis with interstitial pneumonia. The patient had complained of chronic dysphagia and reflux symptoms. A small and asymptomatic diverticulum was first detected four years ago. Endoscopy repeated because of exacerbation of symptoms revealed an enlarged diverticulum. Therefore, the patient underwent laparoscopic diverticulectomy and Heller myotomy with partial fundoplication. Her postoperative course was uneventful, and her symptoms were relieved.

**Clinical discussion:**

Although patients with systemic sclerosis commonly present with reflux esophagitis, they rarely develop achalasia-like change that leads to an esophageal diverticulum. There are several treatment options for esophageal diverticulum, including transhiatal surgery, thoracic surgery, or endoscopic treatment.

**Conclusion:**

Clinicians must pay attention to patient symptoms because the worsening of dysphagia might suggest an underlying achalasia-like change or epiphrenic diverticulum in the esophagus. Surgeons should determine the treatment approach with considerations of the patient's background, the location and size of the diverticulum, and other factors.

## Highlights

• Esophageal epiphrenic diverticulum in patients with systemic sclerosis is extremely rare.
• We report a laparoscopically resected case of esophageal epiphrenic diverticulum with systemic sclerosis.
• Patients with systemic sclerosis can rarely develop achalasia-like change and epiphrenic diverticulum in the esophagus.
• The treatment approach should be determined with consideration of the patient's background and the status of the lesion.

## 1 Introduction

Esophageal epiphrenic diverticulum is categorized as a pulsion type of diverticulum and is a relatively rare condition. An epiphrenic diverticulum is located at the lower esophagus and is found more commonly with esophageal motility disorders such as achalasia or diffuse esophageal spasm [1]. Surgical treatment is indicated for an enlarged or symptomatic diverticulum and concomitant malignancy in the diverticulum. Systemic sclerosis (SSc) is a disease characterized by autoimmune inflammation, fibrosis of the skin and internal organs, and vasculopathy [2]. Gastrointestinal (GI) involvement has been estimated at nearly 90 %, and a diverticulum is one of the common features in SSc [3]. A diverticulum is found most often in the small or large intestine of SSc patients, but an esophageal epiphrenic diverticulum is extremely rare [4]. We present a case of an esophageal epiphrenic diverticulum with SSc-related esophageal motility disorder treated with laparoscopic diverticulectomy and Heller myotomy.

This work was reported in line with the SCARE 2020 guidelines [5].

## 2 Case presentation

A 73-year-old woman had been treated for diffuse SSc with interstitial pneumonia by prednisolone 5 mg per day in the Department of Internal Medicine. Blood tests were positive for anti-Scl-70 antibody and negative for anti-centromere antibody and anti-RNA polymerase III antibody. The patient had complained of chronic dysphagia and reflux symptoms. Although annual follow-up with endoscopy had first detected an epiphrenic esophageal diverticulum four years ago, the diverticulum was small and asymptomatic. Endoscopy was repeated because of exacerbation of symptoms and onset of postprandial epigastric pain and revealed an enlarged esophageal diverticulum, for which the patient was referred to our department for surgery.

**Fig. 1:**
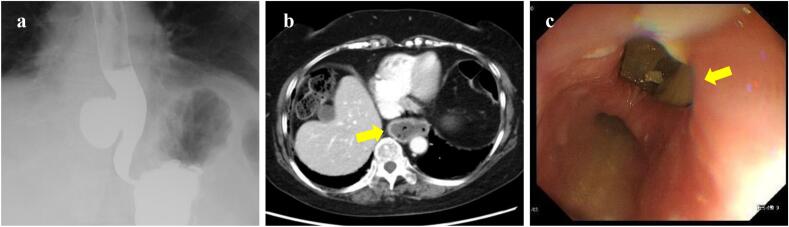
Preoperative findings. a. Contrast esophagography shows a 50-mm epiphrenic diverticulum on the right wall of the lower esophagus. b. Contrast-enhanced computed tomography shows a diverticulum (yellow arrow) at the lower mediastinum. c. Upper endoscopy shows the orifice of the diverticulum (yellow arrow).

Contrast esophagography and enhanced computed tomography revealed a large 50-mm diverticulum in the right wall of the lower thoracic esophagus (Fig. 1a, b). Esophageal dilatation was mild. The diverticulum was located 5 cm above the esophagogastric junction (EGJ). Upper endoscopy showed a diverticulum in the lower thoracic esophagus with internal residue and mild reflux esophagitis (Fig. 1c).

**Fig. 2:**
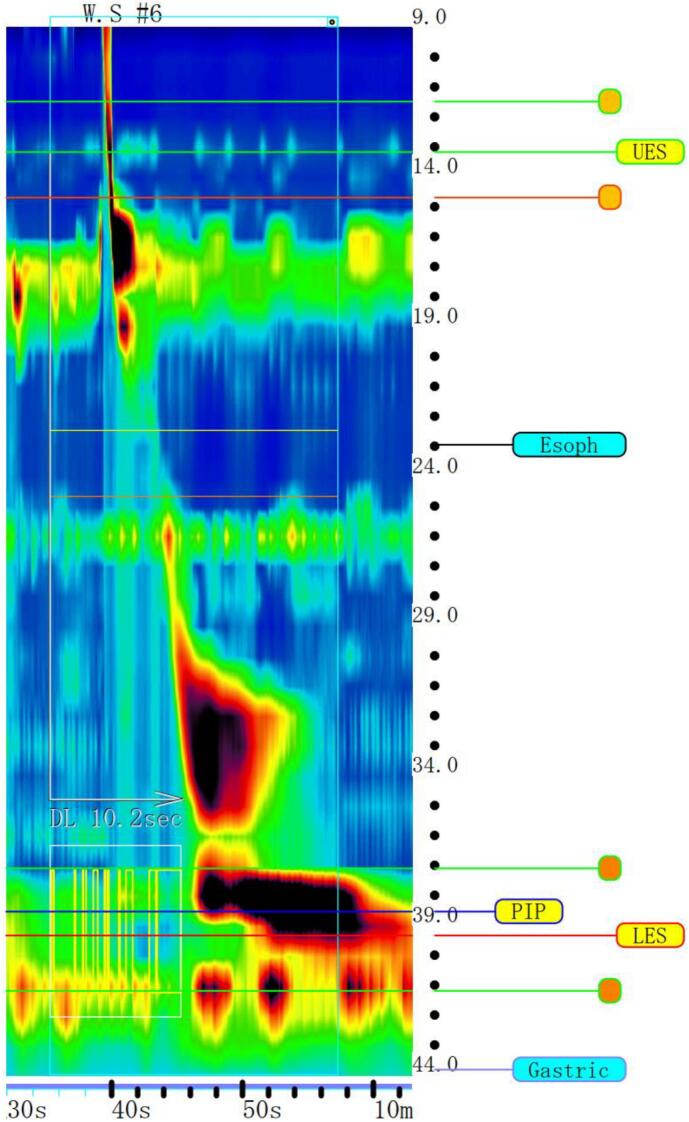
High-resolution esophageal manometry shows persistent high internal pressure in the lower esophagus. Increased internal pressure is also observed in the area consistent with a diverticulum (30–36 cm from the incisors).

High-resolution esophageal manometry (HREM) revealed persistent high internal pressure in the lower esophagus, which was exacerbated during swallowing (Fig. 2). Increased internal pressure was also observed in the area consistent with a diverticulum. As the integrated relaxation pressure was high (median: 41.3 mm Hg) and esophageal peristalsis was observed. This finding was categorized as EGJ outflow obstruction (incompletely expressed achalasia or mechanical obstruction) in the Chicago classification v3.0 [6]. The presence of an esophageal motility disorder associated with SSc was suspected.

**Fig. 3:**
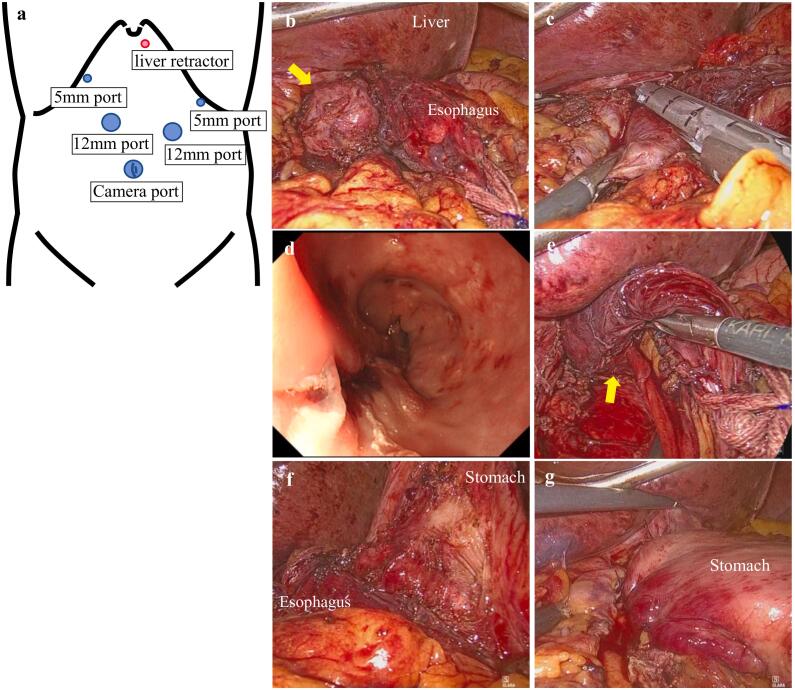
Port placement and intraoperative findings. a. The operator used the two ports on the right side of the patient. b. The diverticulum (yellow arrow) was dissected from surrounding tissues and completely exposed. c. The diverticulum was transected using a linear stapler with endoscopic bougie. d. Intraoperative upper endoscopy showed complete resection of the diverticulum and no stenosis in the esophagus. e. The stapler line was covered and reinforced by suturing (yellow arrow). f. Heller myotomy was performed for over 2 cm from the EGJ on both the esophageal side and gastric side. g. Partial fundoplication and fixation to the diaphragmatic crus were performed.

**Fig. 4:**
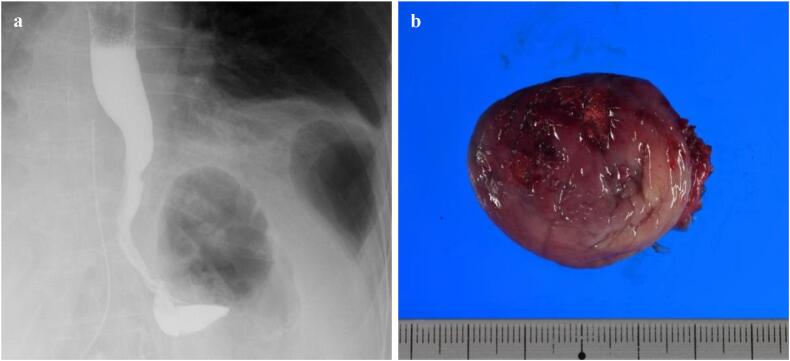
Postoperative images. a. Postoperative contrast esophagography shows smooth flow of contrast medium to the stomach. b. Photo of the specimen.

The patient subsequently underwent laparoscopic diverticulectomy with Heller myotomy and partial fundoplication. We started the laparoscopic procedure with five ports and an additional incision for a liver retractor (Fig. 3a). The patient's tissues were fragile and hemorrhagic due to long-term steroid use. The diverticulum was dissected transhiataly from the esophagus and surrounding tissues in the inferior mediastinum and resected using a linear stapler with endoscopic confirmation (Fig. 3b–d). The stapler line was covered and reinforced by suturing (Fig. 3e). The vagus nerve was preserved. We performed a Heller myotomy on the opposite side of the diverticulum for over 2 cm from the EGJ on both the esophageal side and gastric side (Fig. 3f). The gastric fornix was fixed to the anterior wall of the EGJ (Fig. 3g). The dilated diaphragmatic crus was closed, and then the esophagus and stomach were fixed to the crus. Because of the fragile tissue and concern about leakage and pulmonary complications, we treated the patient more carefully than usual during her postoperative days. On the seventh postoperative day, contrast esophagography showed no evidence of leakage or stenosis and oral feeding was resumed (Fig. 4a). The patient was discharged on the 12th postoperative day. Dysphagia was improved and her epigastric pain had disappeared. The pathological diagnosis of the specimen was a pseudodiverticulum without a muscular layer and with no evidence of malignancy found (Fig. 4b).

## 3 Discussion

Epiphrenic diverticulum is an esophageal diverticulum with a prevalence ranging from 0.015 to 2 % that is caused by esophageal motility disorder including achalasia, diffuse esophageal spasm, or hypertensive lower esophageal sphincter (LES) [1], [6]. Surgical treatment is indicated in cases with worsening symptoms or complications including malignancy. The standard surgical treatment is transhiatal diverticulectomy or Heller myotomy as minimally invasive surgery (MIS) [7]. Nadaleto et al. reviewed 21 reports about MIS for epiphrenic diverticulum [8]. A total of 210 patients who underwent MIS were included in this review, including 180 cases of laparoscopic transhiatal surgery (85.7 %), 24 cases of video-assisted thoracoscopy (11.4 %), 3 cases of combined laparoscopic surgery and video-assisted thoracoscopy (1.4 %), 3 cases of robotic surgery (1.4 %). The question of whether transhiatal or thoracic approach should be chosen has been described in several reports. The advantages of the transhiatal approach are better access to the EGJ and easier performance of the myotomy and fundoplication. However, the thoracic approach is also effective in some cases. Previous studies showed the indications for the thoracic approach to be (1) adhesions in the mediastinum, (2) large size of the diverticulum, (3) location of diverticulum (>5 cm distant from the EGJ), and (4) failure of the laparoscopic approach [9], [10], [11], [12]. The diverticulum in our patient was on the borderline of an indication for transhiatal versus thoracic approach because the diverticulum was relatively large and located at 5 cm distant from the EGJ. As the patient had interstitial pneumonia related to SSc, we chose the transhiatal approach to minimize the risk of pulmonary complications derived from single-lung ventilation. The patient's clinical course was good, and we believed that ours was the best approach for this patient. Although there are still few reports of robotic surgery for epiphrenic diverticulum, robotic surgery may be a promising treatment option because it is applicable to both transhiatal and thoracic approach and has several advantages over conventional MIS including greater visibility, precision, and dexterity [8].

Another treatment approach for epiphrenic diverticulum is endoscopic treatment. The diverticular peroral endoscopic myotomy (D-POEM), which is a septotomy along with LES myotomy, has been reported as an effective treatment for epiphrenic diverticulum. Nabi et al. reported outcomes of D-POEM in 13 patients with epiphrenic diverticulum. Technical success was achieved in 92.3 % of the patients, and clinical success, which was defined by an Eckardt score ≤3 after treatment, was achieved in 84.6 % at a median follow-up of two years [13]. In our patient, we performed surgical treatment for certainty because the patient had SSc and interstitial pneumonia treated with steroid, and the risk of complications was considered high.

SSc is a rare disease with a prevalence ranging from 3.8 to 50.0 cases per 100,000 individuals [14]. It is characterized by autoimmune inflammation, vasculopathy, and fibrotic tissue disposition leading to loss of function of the skin and internal organs [2]. In patients with SSc, GI involvement has been estimated in nearly 90 %, and up to 50 % of patients have GI symptoms [3]. The esophagus is the most affected area of the GI tract. Patients can suffer from dysphagia, reflux, and heart burn [4]. These symptoms are results of reduced LES pressure, presence of reflux esophagitis, hiatal hernia, or low peristalsis in the esophagus [15]. Diverticula in the intestines are also a common feature of GI involvement in SSc. However, an epiphrenic esophageal diverticulum associated with SSc is an extremely rare condition. Although Ingegnoli and Schioppo reported a Zenker diverticulum in a patient with SSc [16], there are no reports of epiphrenic diverticulum associated with SSc to the best of our knowledge.

As above, esophageal motility disorder associated with SSc mostly shows a low LES pressure in the esophagus, and patients present with reflux esophagitis or hiatal hernia. However, conditions related to hypertensive LES such as achalasia or achalasia-like change have been reported in SSc patients. In a study of HREM in 112 patients with SSc, one patient each had type II achalasia, hypercontractile esophagus, EGJ outflow obstruction, and distal esophageal spasm [17]. Although the mechanism of achalasia-like change in the esophagus of SSc patients is unclear, Woo et al. noted that autoantibody to the myenteric plexus may be involved in this condition [18]. As this antibody has been correlated with the presence of Reynaud's phenomenon in SSc patients and detected in both SSc and achalasia patients, autoimmunity derived from this antibody might lead to achalasia-like change in the esophagus [19], [20]. In our patient, HREM showed persistent high pressure in the lower esophagus. We therefore considered SSc-related achalasia-like change to be present in her esophagus and performed a Heller myotomy and partial fundoplication.

## 4 Conclusion

We presented a rare case of esophageal epiphrenic diverticulum with SSc. Laparoscopic diverticulectomy and Heller myotomy were safely performed. Patients with SSc can rarely develop achalasia-like change that leads to an epiphrenic diverticulum. Clinicians must pay attention to patient symptoms because the worsening of dysphagia might suggest underlying achalasia-like change or an epiphrenic diverticulum in the esophagus. Surgeons should determine the treatment approach with considerations of the patient's background, the location and size of the diverticulum, and other factors.

## Abbreviations

SSc: Systemic sclerosis
GI: Gastrointestinal
EGJ: Esophagogastric junction
HREM: High-resolution esophageal manometry
LES: Lower esophageal sphincter
MIS: Minimally invasive surgery
D-POEM: Diverticular peroral endoscopic myotomy

## Consent for publication

Written informed consent was obtained from the patient for the publication of this case report and accompanying images. A copy of the written consent is available for review by the Editor-in-Chief of this journal on request.

## Provenance and peer review

Not commissioned, externally peer reviewed.

## Ethical approval

This case study is exempt from ethical approval.

## Funding

This research did not receive any specific grant from funding agencies in the public, commercial, or not-for-profit sectors.

## Guarantor

The guarantor is Yoshihiro Tanaka.

## CRediT authorship contribution statement

Study conception and design: RA and YT. Acquisition of data: RA, YT, YS, SF and ME. Analysis and interpretation of data: RA, YT, YS. Drafting of manuscript: RA. Critical revision: RA, YT, and NM. Supervision: YT and NM.

## Declaration of competing interest

The authors declare that they have no competing interests.
